# *Chromobacterium violaceum* causing disseminated soft tissue and pulmonary abscesses in a traveller returning from the Azores

**DOI:** 10.1099/acmi.0.000251

**Published:** 2021-08-12

**Authors:** Melissa Chowdhury, Nathaniel Lee, Emmanuel Q. Wey

**Affiliations:** ^1^​ Royal Free Hospital, Royal Free Foundation NHS Foundation Trust, London, UK; ^2^​ Centre for Clinical Microbiology, Division of Infection and Immunity University College London, London, UK

**Keywords:** antimicrobials, clinical microbiology, epidemiology, soft tissue infections, tropical diseases

## Abstract

This case report describes a 30-year-old male patient presenting with *Chromobacterium violaceum* cutaneous lesions who develops a subsequent bacteraemia, complicated by soft tissue and pulmonary abscesses. *C. violaceum* disease is a rare infection that can manifest in a spectrum from cutaneous lesions to disseminated disease and sepsis, the latter associated with high mortality. Although in the available literature there is a recommendation for a prolonged antibiotic course, we describe effective management with a shorter course of antibiotics. This case highlights the importance of not only considering a diagnosis of *C. violaceum* if there has been a high risk and appropriate exposure, but to also consider the changing epidemiology of the organism due to certain geographical areas becoming warmer due to climate change.

## Introduction

With rare infections, clinicians are guided by local epidemiology in order to consider reasonable causative organisms. With the increase in travel and migration, a thorough travel history can often be key to making a diagnosis. However, with global temperatures increasing each year, our idea of where certain organisms are usually isolated may be changing. The following case describes a patient with *Chromobacterium violaceum* infection, an organism traditionally thought to occur within tropical and subtropical climates. With our patient having travelled back from the Azores, a location not within the subtropical latitudes, we illustrate how our diagnostic thought processes may need to keep up with the changes in climate worldwide.

*C. violaceum* is a Gram-negative facultative anaerobic, motile, oxidase-positive bacillus [1]. It was first described in 1872 as a tropical pathogen that was potentially harmful to people and animals [2]. It produces a characteristic dark violet antioxidant pigment called violacein, which produces a purple colouring in colony growths [3]. It is a rare infection with fewer than 300 cases reported in the literature [4]. *C. violaceum* disease typically presents with skin or soft tissue infections that can rapidly progress to multiorgan failure. It had been historically considered to be an organism typically found only in tropical and subtropical countries [5]. It is found in aquatic environments and is sensitive to temperature [1]. Infections have mainly occurred in the western Pacific, southeast USA and south-east Asian regions [6]. Cases described in Europe were presumed to be imported from tropical regions [6].

Having a high index of suspicion for this infection is vital as it is associated with high mortality. To prevent relapses it is mostly recommended for patients to have a long duration of antibiotics [1]. Our case, however, illustrates that this may not necessarily always be needed. As it is a rare infection with few case reports, it may be that the more serious infections that have occurred were more likely to be published. This is of particular importance since one of the recommended treatments is with a fluoroquinolone. Considering shorter courses will therefore help to minimize exposure to the potentially harmful side effects of this antibiotic class.

## Case report

A 30-year-old presented to the emergency department with an 8-day history of widespread pustular follicular lesions, particularly concentrated in the axillary and anterior torso regions. The patient had just returned from a 2-week trip with his wife to the Azores, Portugal, 1 day before the rash appeared. The day before the patient’s return he had visited a hot spring on Sao Miguel Island.

Six days prior to hospital admission, most of the patient’s lesions had subsided and resolved except for three sites: one in his right axilla and two lesions in the right lower abdominal area. Over the subsequent days, these began to increase in size, most notably the right axillary lesion. One day preceding hospital admission the patient became systemically unwell with fevers, vomiting and anorexia. His only significant past medical history was discoid lupus managed with topical steroids. He worked as a health economist, was a non-smoker and had no history of recreational drug use.

On examination at admission, he was pyrexial (37.7 °C) and tachycardic (heart rate 135 min^−1^) with a blood pressure of 112/66 mmHg. His respiratory rate was 25 breaths min^−1^ and his oxygen saturation was 98 % on room air. He was noted to have a fluctuant, tender and warm mass in his right axilla measuring 8×3 cm, as well as less-defined fluctuant mass in his suprapubic region with overlying erythematous skin.

His admission complete blood count showed results of haemoglobin 125 g l^−1^ and total WBC 35.31 10^9^ l^−1^, with an absolute neutrophil count of 30.47 10^9^ l^−1^. His lactate was 2.04 mmol l^−1^, his eGFR was 69 ml min^−1^ and his C-reactive protein was 338 mg l^−1^. His admission blood culture did not have any growth after 48 h and 5 days’ incubation. An HIV screening test was negative. A methicillin-resistant *Staphylococcus aureus* skin swab screen was negative.

**Fig. 1.**
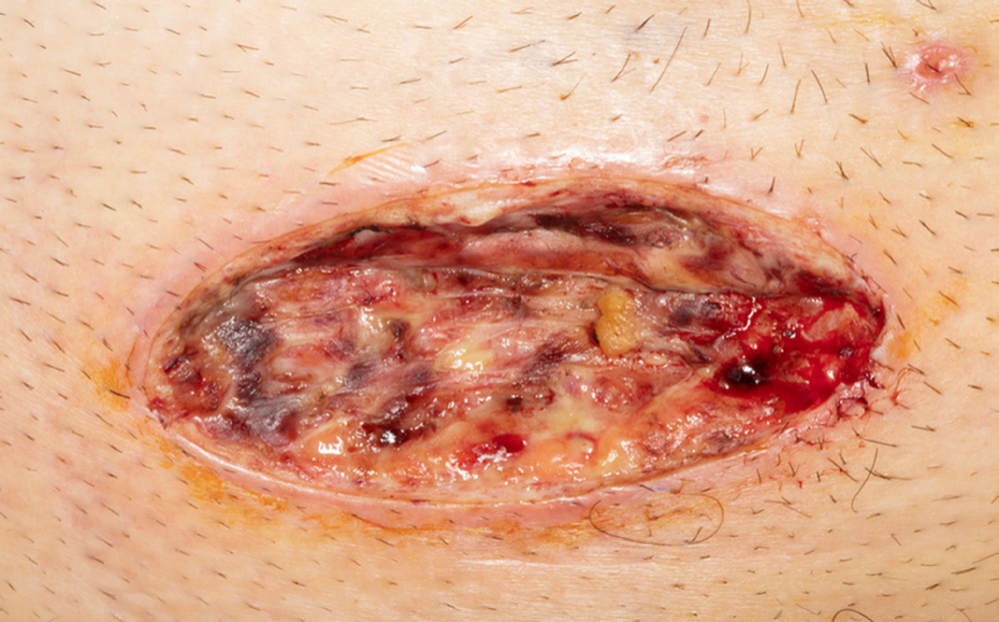
Incised abscess located on abdomen. Note smaller inflamed lesion nearby.

He was admitted under the general surgeons with a diagnosis of systemic sepsis secondary to bacterial abscesses. He was started empirically on intravenous amoxicillin/clavulanic acid. A surgical incision and drainage was performed on his abdominal and axillary lesions on the day of admission, with large quantities of pus being drained, which was sent for microscopy and culture. A macroscopic image of the incised abdominal lesion is presented in Fig. 1.

The pus fluid obtained from both surgical sites was set up and incubated on blood agar (BA), chocolate blood agar (CBA) and a cysteine–lactose–electrolyte-deficient agar (CLED). No organisms were seen on auramine staining, and the samples were analysed on the GeneXpert platform and found to be negative for *Mycobacterium tuberculosis *. After 24 h incubation, smooth, round and convex colonies were noted on the CBA plate. A subset of colonies was processed using matrix-assisted laser desorption/ionization time-of-flight mass spectrometry (MALDI-TOF MS) (Bruker, USA), which reported a result of *C. violaceum* with a modified score value of 1.86, indicative of identification to genus level.

Post-operatively he was transferred back to a ward environment. Day 1 (D1) post-admission he developed persistent fevers (>38 °C) and became increasingly hypotensive, tachycardic and oliguric despite adequate fluid resuscitation. A CT scan taken at the time confirmed the presence of multiple soft tissue nodes and pulmonary nodules, as well as complications related to systemic sepsis, such as bilateral patchy basal consolidation. His antimicrobials were switched to piperacillin/tazobactam and intravenous clindamycin. He was taken for a surgical washout of his lesions and then transferred to the intensive therapy unit (ITU) for haemodynamic support with ionotropes.

D2 post-admission he was reviewed by the Infectious Diseases team on the ITU, with the results of the initial laboratory analysis suggestive of *C. violaceum *. Based on the organism identification, the empirical antimicrobial treatment regimen was changed to meropenem, intravenous ciprofloxacin and gentamicin.

**Fig. 2.**
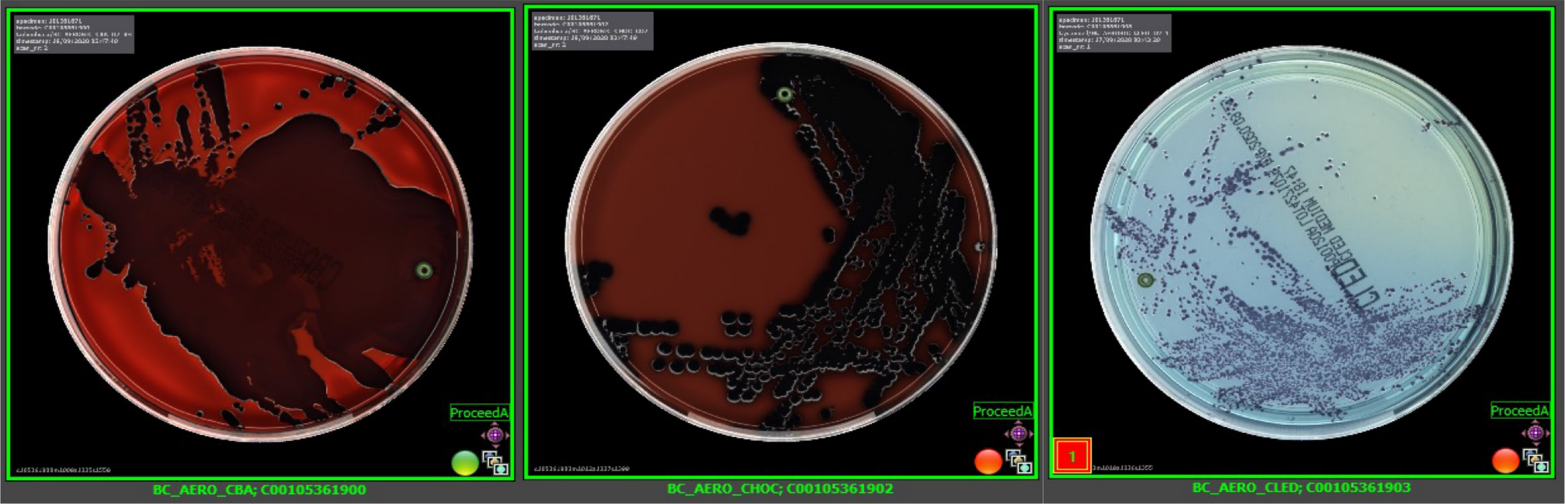
*
C. violaceum* growth on blood agar, chocolate blood and cysteine–lactose–electrolyte-deficient agar.

**Fig. 3.**
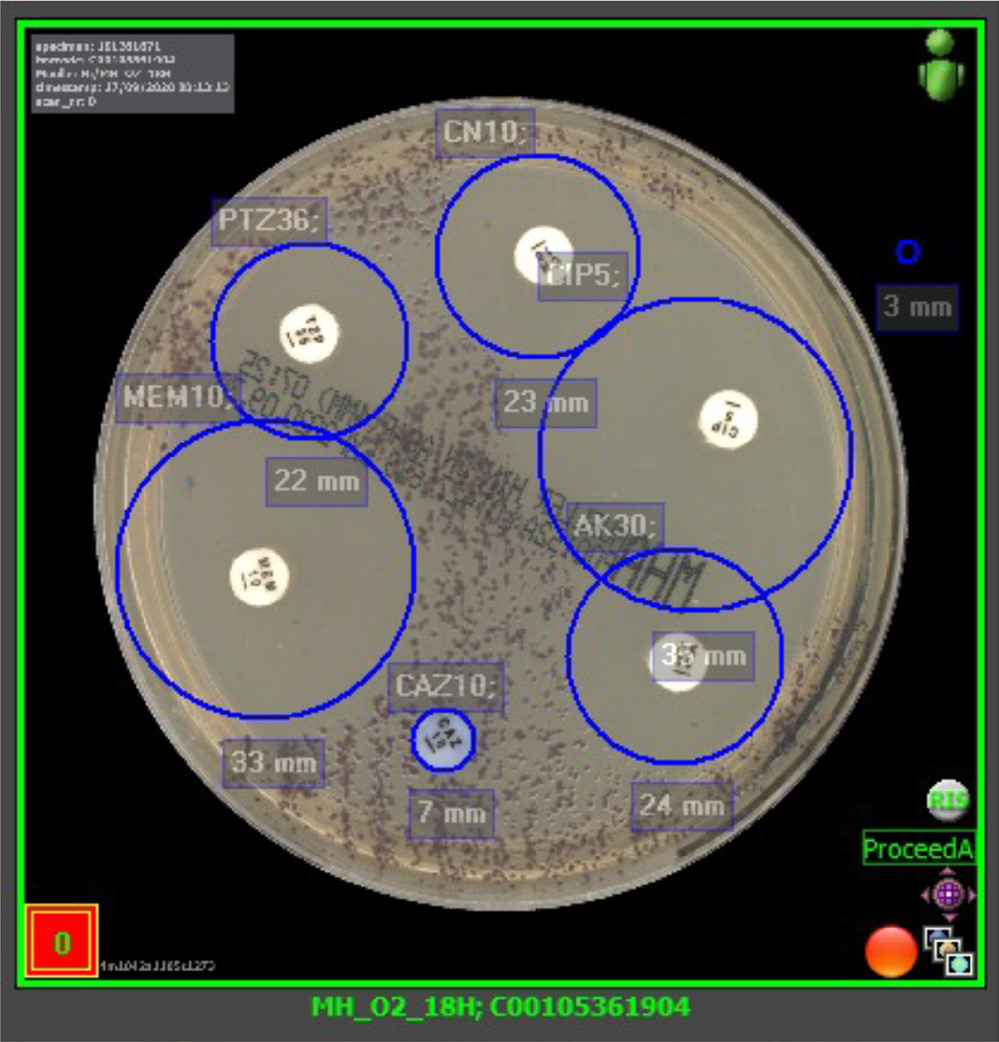
Antimicrobial sensitivity testing by disc diffusion.

Further microbiological tests and results were available at this point. A legionella urinary antigen test was negative. A peripheral blood culture taken on D1 post-admission had flagged positive on the incubator with an organism identified via MALDI-TOF as a *Chromobacterium* spp. Growth plates of the initial pus sample are shown in Fig. 2. Antimicrobial susceptibility testing on the initial pus samples using a disc diffusion method was employed (Fig. 3). Discs on Mueller–Hinton agar were set up for ceftazidime, gentamicin, meropenem, ciprofloxacin, piperacillin/tazobactam and amikacin using the European Committee on Antimicrobial Susceptibility Testing (EUCAST) breakpoints for *Pseudomonas* spp.

The patient improved following the switch of his antimicrobials. He was stepped down to the general surgical ward on D5 post-admission. Antimicrobial disc sensitivities reported good zone diameter sizes for meropenem (33 mm) and ciprofloxacin (35 mm), and so all of his antimicrobials were discontinued except for meropenem. A transthoracic echocardiogram did not show any evidence of valvulopathy. His clinical state, mobility and appetite improved over the next week and he was discharged on D12 with a course of oral ciprofloxacin 750 mg twice daily to complete a total of 6 weeks of effective antimicrobial use.

Subsequent final microbiological tests were completed in the interim. Extended fungal cultures after 3 weeks did not yield any growth, and TB cultures on tissue and pus samples from the abscesses were negative. The final report returned from the national reference laboratory (BRD, PHE Colindale) indicated a species ID of *C. violaceum* and the following MICs: ciprofloxacin (4 mg l^−1^), ceftazidime (4 mg l^−1^), imipenem (2 mg l^−1^), meropenem (2 mg l^−1^) and piperacillin/tazobactam (4 mg l^−1^).

## Discussion

*C. violaceum* typically presents with skin or soft tissue infections that can rapidly progress to multiorgan failure. Associated complications include abscesses in visceral organs, including the liver, lung, brain and spleen [4]. Predisposing conditions appear to be chronic granulomatous disease, glucose-6-phosphatase deficiency, and diabetes [1, 7]. It is an infection slightly more common in men (reported in some literature as 55.6 %), and which usually affects people between the ages of 16–66 years of age [3]. Contact with contaminated fresh water is also a documented associated risk factor, which reflects swimming in outdoor hot springs in our case [8].

The last decade contained several years that were documented as the warmest on record [9]. With the evidence for climate change mounting, the impact on the environment and global distribution of infections must be considered. The effect of temperature at the micro-organism level is unknown. Previous studies have looked at *C. violaceum’s* proteomic signatures in response to stress and have demonstrated its ability to adapt expression of cellular structures to these external signals [10]. Upregulation of certain virulence factors, such as the type III secretion system, may be predictive of virulent strains with the capacity to infect humans despite the organism’s evolution as a free-living environmental bacterium [11].

The historical extent of global territory for *C. violaceum* has been considered to be between latitudes of 35 degrees north and 35 degrees south [1]. However, several severe *C. violaceum* infections have now been documented that originated from areas beyond these latitudes [1]. The effects of climate change cannot be discounted as a contributory factor, not only in terms of warming temperatures, but also the associated extreme weather conditions that promote flooding [12]. As clinicians, we need to be mindful of this and be able to adapt our clinical practice accordingly.

It is important for *C. violaceum* infections to be promptly recognized and treated as mortality outcomes of ~50 % have been reported [6]. The major risk factors for mortality are young age, the presence of abscesses, shorter duration of clinical course and inappropriate antimicrobial therapy [1]. Clinical and Laboratory Standards Institute (CSLI) and EUCAST breakpoints are not available for *C. violaceum *. In our reported case, antimicrobial susceptibilities were interpreted using *Pseudomonas* spp. susceptibility criteria. In terms of treatment, there have been reports of successful treatment either with ciprofloxacin alone, or in combination with piperacillin/tazobactam or trimethoprim/sulfamethoxazole [13–15]. The support for a second agent has been due to reported resistance to ciprofloxacin [15]. There are, however, currently no trials comparing appropriate antibiotic therapy for this infection.

Within the available literature, prolonged treatment is recommended, with treatment being continued up to 3 months in successfully treated cases [8, 16]. In some instances, shorter courses resulted in clinical improvement, but were associated with fatal relapses [17]. A hypothesis for this is that occult microabcesses or hidden septic foci may persist despite appropriate treatment, and a 2–4 week period of parenteral antibiotics followed by maintenance therapy with an oral agent for 2–3 months is recommended by some [1].

This contrasts with the clinical course of our case, who received 1 week of meropenem and 6 weeks in total of ciprofloxacin, and was followed up closely. The patient had a dramatic response to treatment and has so far not had a relapse of infection. This might be explained by early identification of the organism and targeted treatment. However, the difference in clinical outcome might also be due to the lack of available literature detailing different clinical outcomes.

*Lin et al.*’s 2016 review of *C. violaceum* infections in Australia reported an overall mortality rate that was much smaller at 7.1 % [18]. It is possible that due to few cases being reported, with the serious cases more likely to be published, the reported mortality may be inflated [18]. Additionally, this review looked at all laboratory isolates of *C. violaceum* in one location, and identified cases that were not associated with clinical disease, suggesting that there may be asymptomatic colonization of *C. violaceum* in humans [18].

With this in mind, early targeted therapy rather than a long duration of antibiotics may be what is required to treat some *C. violaceum* infections. As in our case, this approach avoids a lengthy exposure to fluoroquinolones with its associated side effects.

## Conclusion

We present a case of disseminated *C. violaceum* with clinical features of soft tissue and pulmonary abscess. Due to changing global climate, the distribution and incidence of this disease is likely to increase. Care should be taken to consider the diagnosis in returning travellers who have had contact with natural thermal baths, particularly from certain geographical distributions.
